# Only a Vine-Slip

**Published:** 1881-04

**Authors:** T. G. Appleton


—*Popular Science Monthly.*

### ONLY A VINE-SLIP.

BY T. G. APPLETON.

The world's life is one long day, in which events strike the hours. The hours strike, time moves on, till another stroke marks an advance which could only be that of the present moment, and to which all the minutes of the past have contributed. I seemed to hear that clock of the ages strike, the other day at my table, when a charming young Frenchman was explaining why he had come to America. It is humiliating to man, haughty in his mastery of this world to find a successful antagonist, the least of creatures, microscopically small, and nameless often, till the baffled husbandman is obliged to write it in Greek or Latin characters upon the banner of his conquering foe. The *Phylloxera* is mightier than a German army; for the latter, once satiated, goes home, but the former apparently stays forever. The Egyptians are again upon us; the plagues of Egypt, and perhaps, what is worse, the plagues of America, move across the world, devastating as they go. The contagion of evils seems to outrun that of beneficence in an unfair proportion. Creatures unconscious of what they do, which the microscope barely discovers, terrify whole nations, and give the lie to the arrogance of man. Somewhat as the old thought is now superseded, the old faith of the mind sapped, by the maggot in the brain which breeds doubt and denial, so in these latter days, the old beldam Earth breeds *oidium* which blights, the phylloxera which destroys. It was the phylloxera which interested my young Frenchman. He had just come from California, after sending home countless *boutures* (vine-slips) to his father, a *vigneron* of Burgundy. It is found that these bits of vine, planted in France and then grafted with the vine of the district, will resist in most cases the phylloxera, and so save the vineyard. It was when hearing this from my young *vigneron* that I seemed to hear that earth-clock strike. The sound I thought was braided of two murmurs where joy and sorrow blended. "Yes," I said to myself, "youth is a good thing, and how beautiful it is to see it sustaining the decrepitude of age!" How proud, thought I, should America be to see her democratic blood mingled with to sustain the princely lives which Bacchus honors! American girls wear the strawberry-leaf and sit not far from the throne itself. And how like this is the marriage between *parvenus* of California and those princely ones whose *etiquette* gleams at royal boards! But how are the mighty fallen! The imperial house of Clos Vougeot is in the dust, and many another lordly house besides. A friend of mine, an expert in the science of wine, crowned his wealth by the purchase of this imperial Clos (field) of Vougeot. This little field, the most precious for its extent in France, a true Field of the Cloth of Gold, whose grape is the highest expression of God's beneficence through the vine He gave us, has a flavor, perfumed, modest, tasting of the violet, which separates it from the crude and harsher vintages as a gentleman is distinguished among roughs. This favorite of the earth this consummate flower of France, Providence shall not long allow to lie perishing in the dust. And it is the democrat who shall fly to the rescue of this scion of an imperial house. For the world of epicures will not be deprived of its dainties; and it is no more than justice that America should heal the wound she makes, for it is confidently asserted that vines from America, imported into the south of France, brought the phylloxera with them. But this is only guess-work; there is a mystery in the modern sudden distribution over the world of insects and weeds which is not understood. It cannot be watched, because it is not suspected, and secretes itself as part of a freight fetched for quite another purpose. Mrs. S. C. Hall has told us how, for ten years before, the weed *Anacharis alsanastrum* spread with frightful rapidity over the inland waters of England, choking ponds and rivers, as may be seen in the Serpentine of London, the germs of which plant were supposed to have been secreted in imported timber. I have recently, however, read that this plant was dying out, apparently finding its environment unsatisfactory. It is interesting also to hear of a process, the reverse somewhat of California's grape-cure, namely, how the robust weeds of England devour, as Britons do the natives, the weaker weeds of Australia. So have we devoured and displaced the Indians; and some think the plucky English sparrow, after whipping all birds of its weight, is destined in the future to take their place. The law which permits such strange invasions of foreign seeds and insects is evidently not a demand for them in a place where they strike. It is no part of a scheme of use or beneficence; they are a law unto themselves, and attach where they can find a foothold. They ravage as a fire does where the air or the earth is ready to flame like tinder, and they challenge man, as yet victoriously, to match his newly acquired knowledge, his microscope, and his chemistry, with the vivacity of their attack. The renowned M. Pasteur vainly fights for his silk-worm, nor is the battle yet decided; soon all the resourses of science, all the skill of the naturalist, will be needed to beat back the invading armies. Man has made, in so much, Nature his slave. He bridges the ocean, he pierces the Appian barrier, he trains the lightning to fetch and carry for him, he pierces stellar space, he analyzes the sun, and, when he feels an obstacle, he forces his way; neither the sands of Suez nor the marshes of Panama delay him; but he stands baffled before his invisible enemy. A breath of air can poison his cities, or devastate his harvests. Nature thus comes back with an unexpected boomerang. *Entre nous, deux maintenant.* And, though proud man clearly anticipates his final triumph, cholera and yellow fever come and go at their own sweet will. The Colorado beetle journeys comfortably to visit foreign parts. A new plague of locusts, the grasshopper of the West, leaves famine behind him as he moves, and even the Gascon wine, into which the nose of Thackeray once dipped, feels an unholy presence, the blight of a new disease.

But there are other things which fly on the wings of the wind besides the seed capsule which nourishes, the flower-germ which ornaments; thought also goes with them as they fly. Never was it so volatile, not as once hoarded in some vast brain, packed into folios by the weary hand, buried in the silence of cloisters through long ages, and at last fructifying suddenly in a thousand lives. Thought now is ever active, omnipresent; an idea now germinates in San Francisco, and a week later is stale in India. The solidest creeds get their edges frittered away by these clouds of passing thought. Custom secures a little the stability of things, but much of it is automatic—the heart is gone out of it. The monarchist is often a better democrat than his American brother, the priest who intones his service is tampering with agnostic infidelities, the Arab sheik present you with his photograph, and the King of Siam adds a lift to his palace. There is hardly such a thing as discussion; assertion usurps the hour, and there is no reply. Everybody could be on either side of questions which once distracted the world; and as we see the Prince of Wales over a cigar trifling with democratic notions, the old noble humbled before the feudality he represents, the priest meshed in a network of hypocrasies from which he would gladly escape—when we see these things, are we not reminded of the phylloxera which can taint the goblet in the hand of a king, of the secret foe which silently saps the health of cities, and that confusion of life and death, which, flying upon the wings of the wind, affronts the stablest security and makes a jest of human conservatism?—*The Popular Science Monthly.*

